# Subcellular localisation of FLAG tagged enzymes of the dynamic protein S-palmitoylation cycle of *Trypanosoma cruzi* epimastigotes

**DOI:** 10.1590/0074-02760180086

**Published:** 2018-05-28

**Authors:** Cassiano Martin Batista, Felipe Saad, Stephane Pini Costa Ceccoti, Iriane Eger, Maurilio José Soares

**Affiliations:** 1 Fundação Oswaldo Cruz-Fiocruz, Instituto Carlos Chagas, Laboratório de Biologia Celular, Curitiba, PR, Brasil; 2 Universidade Estadual de Ponta Grossa, Departamento de Biologia Geral, Ponta Grossa, PR, Brasil

**Keywords:** dynamic S-palmitoylation, Trypanosoma cruzi, protein expression

## Abstract

Dynamic S-palmitoylation of proteins is the addition of palmitic acid by zDHHC palmitoyl transferases (PATs) and depalmitoylation by palmitoyl protein thioesterases (PPTs). A putative PAT (TcPAT1) has been previously identified in *Trypanosoma cruzi*, the etiological agent of Chagas disease. Here we analyse other 14 putative TcPATs and 2 PPTs in the parasite genome. *T. cruzi* cell lines expressing TcPATs and TcPPTs plus a FLAG tag at the C terminus were produced for most enzymes, with positive detection by indirect immunofluorescence. Overexpressed TcPATs were mostly found as single spots at the parasite anterior end, while the TcPPTs were dispersed throughout the parasite body.

Dynamic protein S-palmitoylation concerns the addition of palmitate to cysteines of the modified protein by zDHHC palmitoyl transferases (PATs) through thioester linkages and depalmitoylation by palmitoyl protein thioesterases (PPTs) (Conibear and Davis 2010). Protein S-palmitoylation cycles promote the insertion of target proteins into membranes, regulating their localisation and function (Linder and Deschenes 2003).

PATs are key transmembrane enzymes, with cysteine rich domains in the DHHC motif (CRD-DHHC domain), in addition to DPG and TTxE structural domains (Greaves and Chamberlain 2011). PATs are involved in diverse biological processes in several organisms, such as *Homo sapiens* cancer (Ducker et al. 2004) and neurological diseases (Young et al. 2012, Cho and Park 2016), yeast endocytosis (Feng and Davis 2000), *Cryptococcus neoformans* virulence (Santiago-Tirado et al. 2015), *Giardia lamblia* encystation (Merino et al. 2014) and invasion in Apicomplexa (Frénal et al. 2013). TbPAT7 is responsible for flagellar localisation of calflagin in the trypanosomatid protozoan *Trypanosoma brucei* (Emmer et al. 2009).

PPTs belong to the serine hydrolases family, are less abundant in number than PATs and are characterised by the presence of a serine active site for hydrolysis of the substrate, being able to cleave amide, ester and thioester bounds (Long and Cravatt 2011).

Goldston et al. (2014) identified, by *in silico* search, 15 PATs in the *Trypanosoma cruzi* genome, as opposed to 12 in *T. brucei* and 20 in *Leishmania major*. However, up to now only one PAT has been characterised in *T. cruzi*, the etiological agent of Chagas disease: TcHIP, or TcPAT1 (Batista et al. 2013). TcPAT1 is a 95.4 kDa Golgi protein expressed in different developmental stages of the parasite, with a modified DHYC motif (Batista et al. 2013). Such modified motif is functional in the homologue Akr1p enzyme of *Saccharomyces cerevisae* (Roth et al. 2002).

It has been recently shown that dynamic protein S-palmitoylation is involved in life cycle progression and virulence in some pathogenic protozoa (Brown et al. 2017). However, no evidence of global PATs or PPTs expression has been yet reported in *T. cruzi*. Thus, aim of this work was to verify the expression of dynamic protein S- palmitoylation enzymes in *T. cruzi* Dm28c (Contreras et al. 1988) epimastigote forms. An *in silico* search for PATs was made in the *T. cruzi* genomic data base (TritrypDB), in parallel with nucleotide BLAST alignment (nBlast-NCBI, Bethesda, MD, USA) of *T. cruzi* genes with the well characterised *S. cerevisiae* PAT genes that encode for Erf2 (with DHHC-CRD motif) (Lobo et al. 2002) and Akr1p (with DHYC-CRD motif) (Roth et al. 2002). As a result, 15 PATs genes were found, identical to that formerly identified by Goldston et al. (2014). Size of these genes varied from 768 (TcPAT7) to 2610 (TcPAT1) base pairs and the resulting protein products were between 30 and 95.4 kDa. Sequence identity between the TcPATs was very low, between 14.2% and 26.83%, as assessed using multiple alignment with Clustal Omega (EMBL-EBI, Cambridgeshire, UK). The softwares TMHMM Server v. 2.0 (Center for Biological Sequence Analysis, CBS, Lyngby, Denmark) and Phyre2 (Kelley et al. 2015) were used to predict transmembrane regions and calculate 3D protein models, respectively. It could be determined that these proteins had three (TcPATs 2 and 6) to seven (TcPAT5) transmembrane domains. By using pFAM software (Sanger Institute, Cambridge, UK) to predict protein domains, it was found that only TcPAT1 had the DHYC motif, while the number of cysteines close to the DHHC/DHYC motif varied from 5 to 9. Only TcPAT4, TcPAT10 and TcPAT14 had both DPG and TTxE structural motifs. On the other hand, TcPATs 5 and 9 had only the DPG motif, while TcPATs 1, 7 and 8 had only the TTxE motif (Fig. 1).

**Fig. 1:**
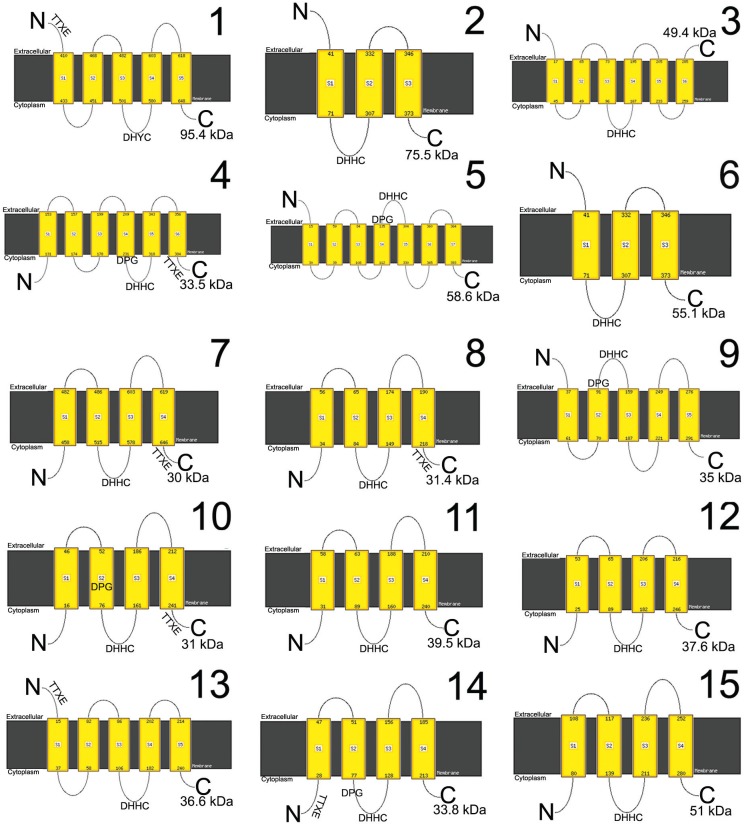
identification and *in silico* analysis of *Trypanosoma cruzi* PATs. Fifteen TcPATs were identified, with predicted molecular mass from 30 to 95.4 kDa. All proteins had transmembrane domains and contained the DHHC motif, with the exception of TcPAT1 (DHYC). S1-SN: membrane crossings; kDa: molecular weight of the predicted protein; TTxE and DPG: structural motifs.

All TcPATs showed similar predicted 3D models, except for TcPAT1 (larger and with ankyrin repeats). TcPPTs 1 and 2 were very different from each other. All 3D models had 100% confidence (Fig. 2).

**Fig. 2:**
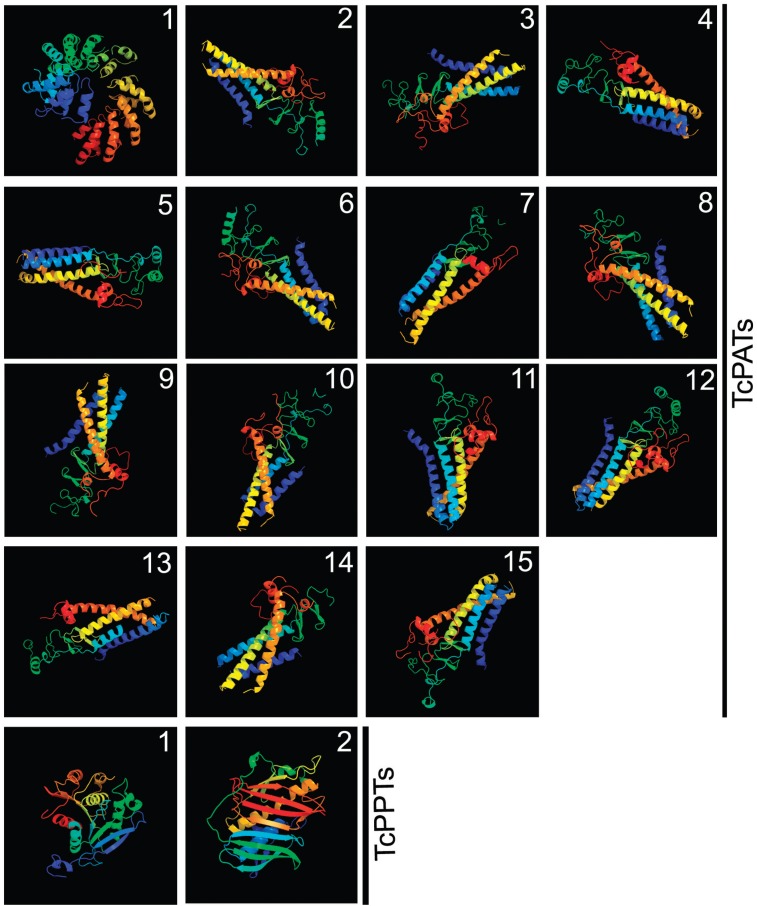
predicted 3D models of *Trypanosoma cruzi* PATs and PPTs. The software Phyre2 was used. All 3D models had 100% confidence.

Aiming to produce transfectant cell lines of *T. cruzi* epimastigotes expressing TcPATs plus a FLAG tag at the C terminus (FLAGC tagged TcPAT), the genes were amplified using specific primers (Table I) with recombination sites for the Gateway cloning platform (Thermo Fischer Scientific, Waltham, MA, USA) by using the entry plasmid vector pDONR 221 and the destination *T. cruzi* vector pTcGWFLAGC (Batista et al. 2010, Kugeratski et al. 2015). All genes were cloned, except TcPAT6 and TcPAT1 (already characterised). Three-day-old *T. cruzi* epimastigotes were transfected with a Gene Pulser XCell BIORAD electroporator (BIORAD Inc., Hercules, CA, USA), selected with 500 µg.mL^-1^ G418 and maintained with 250 µg.mL^-1^ of the same antibiotic, as previously described (Batista et al. 2010). Twelve resistant cell lines could be selected, with the exception of TcPAT4.

**TABLE I:** Primers used for palmitoyl transferase (PAT) isolation from gDNA of *Trypanosoma cruzi* (clone Dm28c) epimastigotes

PAT/Gene ID	F’/R’ (5’-3’)
TcPAT1/*	ATGCAGGTGTTTGGCGCTCGGATG ACGGCGTTCATCTTTCACCT
TcPAT2/ TcCLB.506297.250	ATGCCACAGACTAACAGCACGGAATGG/GGGTTCTCTGACTTCATGCGC
TcPAT3/ TcCLB.510899.50	ATGGGGCCCATACGCGTTGAAAGAG/CACCTGCGTGGCACACAACT
TcPAT4/ TcCLB.508479.200	ATGTCAGGTTTCTGGTCTGTTCAGC/CACCTCTGCTGTTTCAACGACAATAT
TcPAT5/ TcCLB.509029.170	ATGTCCGGAGAGACTTTTGCTTG/CTCATATTTCATCCTCCGTTCTCCT
TcPAT6/ TcCLB.506177.40	ATGCGGTCATCTATGTTGCTGCTTTT/TTCCTCCTTCATCTCCTCCTCGCT
TcPAT7/ TcCLB.510687.130	ATGGATGAATCAAACGACGCG/CACGTCATTCTCAGCGTTTCG
TcPAT8/ TcCLB.511897.19	ATGGGTAAGATTTTTGAAATGGAGGT/CCGTATCAAATCAACAAGAGTTCTCC
TcPAT9/ TcCLB.509769.33	ATGGATTGCGTGGTAGGTATGCGGAAT/AACTAGAGCCTCAGTGTTCAACCAC
TcPAT10/ TcCLB.508239.40	ATGATGTCATTGTTATCACGATGGG/CACAAGGTCGGCGTCATCG
TcPAT11/ TcCLB.511823.50	ATGTCGTTGCTTTGTTGTGATCC/GTCATATTTGGGTGAAATGGGTG
TcPAT12/ TcCLB.506855.10	ATGGGGTCGTTGATTCCGC/CACCGGCAACATCACCTCATC
TcPAT13/ TcCLB.510747.18	ATGAATGTACCCACTTCATCCAGTCCGAT/CACATAAAACTCGGCGTTTTCC
TcPAT14/ TcCLB.511153.60	ATGGAGTCCGTGGAAGTGCTAGT/TGCGATGATGGGGCTCTTATT
TcPAT15/ TcCLB.509105.20	ATGCGGTGCTGTGGGCG/CATACCACCAGATCCGGGAAGCGAC

* Batista et al. (2013); F’: forward primer; R’: reverse primer.

For subcellular localisation by indirect immunofluorescence assays (IFA), *T. cruzi* transfectants were washed twice in PBS, fixed for 10 min with 4% paraformaldehyde, adhered to 0.1% poly-L-lysine coated coverslips, permeabilised with 0.5% Triton/PBS, and incubated for one hour at 37ºC using a mouse anti-flag antibody (Sigma-Aldrich St. Louis, MO, USA) diluted 1:4000 in incubation buffer (PBS pH 7.4 containing 1.5% bovine serum albumin). After three washes in PBS, the samples were incubated in the same conditions with a secondary goat anti-mouse antibody coupled to AlexaFluor 594 (Thermo Fischer Scientific, Waltham, MA, USA) diluted 1:600 in incubation buffer. The samples were washed three times with PBS, incubated for 5 min with 1.3 nM Hoechst 33342 (Sigma-Aldrich St. Louis, MO, USA) and the coverslips were mounted with Prolong Gold antifading agent (Thermo Fischer Scientific, Waltham, MA, USA). The slides were observed in a Nikon Eclipse E600 epifluorescence microscope.

As a result, TcPATs 3, 5, 8, 11, 12, 14 and 15 were located as single dots at the anterior region of the parasite, close to the kinetoplast and the flagellar pocket (Fig. 3). Interestingly, most PATs with four transmembrane domains (five out of seven) showed this pattern. The positive reaction was frequently found lateral to the kinetoplast, which suggests Golgi, flagellar pocket or contractile vacuole localisation. TcPAT2 labeling appeared as strong dots distributed throughout the cell body, suggestive of localisation in some cytoplasmic organelle (Fig. 3). TcPAT13 presented a stronger labeling at the perinuclear region (Fig. 3). These patterns were expected, since PATs are usually found at the endoplasmic reticulum, Golgi and plasma membranes (Ohno et al. 2006). No positive reaction was detected for TcPATs 7, 9, 10 (Fig. 3). Transcriptomic data from TritrypDB indicate that TcPATs 7 and 9 are expressed in metacyclic trypomastigotes, but not in epimastigotes. Therefore, gene expression of these two enzymes (and possibly also TcPAT10) can be down-regulated in epimastigotes. In summary, these results indicated that at least nine TcPATs could be overexpressed in *T. cruzi* epimastigotes.

**Fig. 3:**
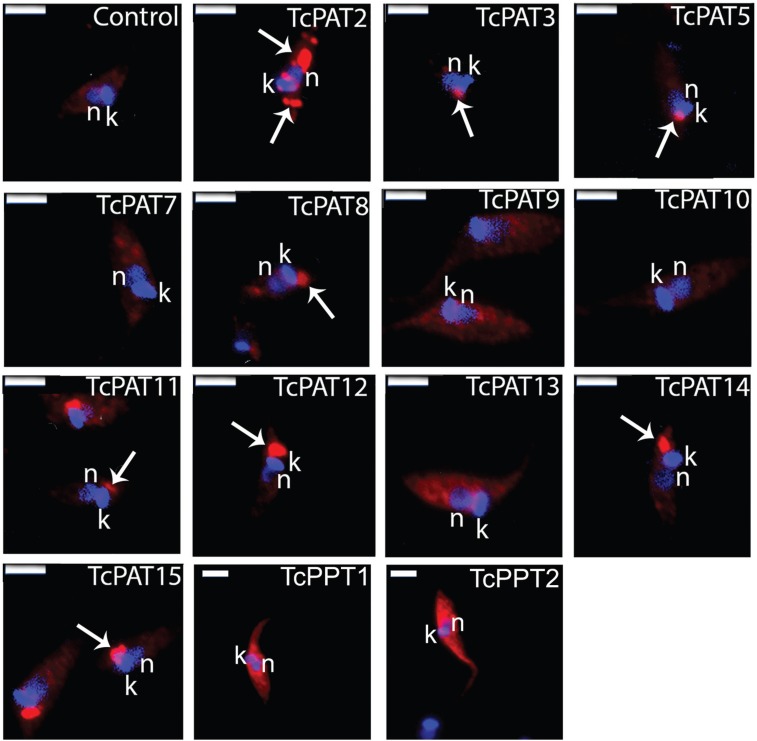
localisation of FLAG tagged PATs and PPTs in *Trypanosoma cruzi* epimastigotes by immunofluorescence assay. Control: wild type epimastigote; blue: hoechst staining of nucleus (n) and kinetoplast (k) DNA; red: PAT (arrow) and PPT labeling with AlexaFluor 594. Bars = 5 µm.

In order to characterise the TcPPTs, a genomic data search was performed as described above, and two genes were identified (Table II). TcPPT1 is an 843 base pairs gene and the product (30.2 kDa) is homologue to *H. sapiens* acyl-protein thioesterase-1 (APT1) and lysophospholipase genes, which are involved in cytosolic and lysosomal protein depalmitoylation (Long and Cravatt 2011). TcPPT2 is a 951 base pairs gene and the product (35.5 kDa) is homologue to *H. sapiens* acyl-protein thioesterase-2 (APT2), involved in cytosolic depalmitoylation (Long and Cravatt 2011). Primers were then designed for isolation and amplification of these genes (Table II).

**TABLE II:** Identification, *in silico* analysis and primer design of *Trypanosoma cruzi* palmitoyl thioesterase (PPT)

PPT/Gene ID	BP	kDa	F’/R’ (5’-3’)
TcCLB.506797.70 (TcPPT1)	843	30.2	ATGATCGGAACGCCGATAGAAAACT/ AGCCTTGGACTCAATCGCCGGCAATACCT
TcCLB.504149.55 (TcPPT2)	951	35.5	ATGCTTCTGCAGGACGTTATTGGAG/ GAGTCTCGATTTGTAGCCCTTTCCTG

BP: number of base pairs; kDa: molecular weight of the predicted protein; F’: forward primer; R’: reverse primer.

The same steps described above for TcPATs were used to produce *T. cruzi* cell lines expressing TcPPTs plus a FLAG tag at the C terminus (FLAGC tagged TcPPTs). Resistant cell lines expressing TcPPT1 and TcPPT2 were selected with 500 µg.mL^-1^ G418. After IFA in the same conditions as described above, both TcPPTs showed strong labeling dispersed through the cell body, suggesting a cytoplasmic localisation (Fig. 3), indicating that *T. cruzi* epimastigotes overexpressed both TcPPTs, in the expected cytoplasmic localisation.

In conclusion, our data indicate that a dynamic protein S-palmitoylation machinery (nine PATS and two PPTs) could be overexpressed in *T. cruzi*. Future studies will be crucial to determine the importance of this machinery for the parasite survival. Palmitoylation and depalmitoylation of proteins can play an important role in this parasite, in events as diverse as nutrition, protein traffic, differentiation, host-cell interaction and infection establishment.
